# Epigenetic Control and Cancer: The Potential of Histone Demethylases as Therapeutic Targets

**DOI:** 10.3390/ph5090963

**Published:** 2012-09-12

**Authors:** Fernando Lizcano, Jeison Garcia

**Affiliations:** Center of Biomedical Research La Sabana University-CIBUS, School of Medicine, Universidad de La Sabana, Campus Del Puente del Común, km 7 Autopista Norte de Bogota, Chía 250001, Colombia; Email: jgarcia@unal.edu.co (J.G.)

**Keywords:** neoplasia, epigenomics, histone demethylases, histone demethylases with a jumonji domain

## Abstract

The development of cancer involves an immense number of factors at the molecular level. These factors are associated principally with alterations in the epigenetic mechanisms that regulate gene expression profiles. Studying the effects of chromatin structure alterations, which are caused by the addition/removal of functional groups to specific histone residues, are of great interest as a promising way to identify markers for cancer diagnosis, classify the disease and determine its prognosis, and these markers could be potential targets for the treatment of this disease in its different forms. This manuscript presents the current point of view regarding members of the recently described family of proteins that exhibit histone demethylase activity; histone demethylases are genetic regulators that play a fundamental role in both the activation and repression of genes and whose expression has been observed to increase in many types of cancer. Some fundamental aspects of their association with the development of cancer and their relevance as potential targets for the development of new therapeutic strategies at the epigenetic level are discussed in the following manuscript.

## 1. Introduction

Cancer is currently recognized as a group of diseases characterized by an abnormal and uncontrolled division of cells, the formation of cellular masses or localized tumors, and the simultaneous formation of independent circulatory and nutrient systems (angiogenesis). Such cells can advance to an invasive-degenerative phenotype in both surrounding and distant tissues through the blood or lymphatic systems via a process known as metastasis, which is the principal cause of death in patients with advanced tumors. Currently, the different types of cancer that affect human beings are considered to be a worldwide public health problem, affecting nearly 13 million people and causing the deaths of eight million people annually [1]. Among the main molecular characteristics that precede the development of this disease are variations in gene expression and genomic instability; these variations are a consequence of disturbances in the processes that regulate protein expression at the genetic and epigenetic levels.

**Figure 1:**
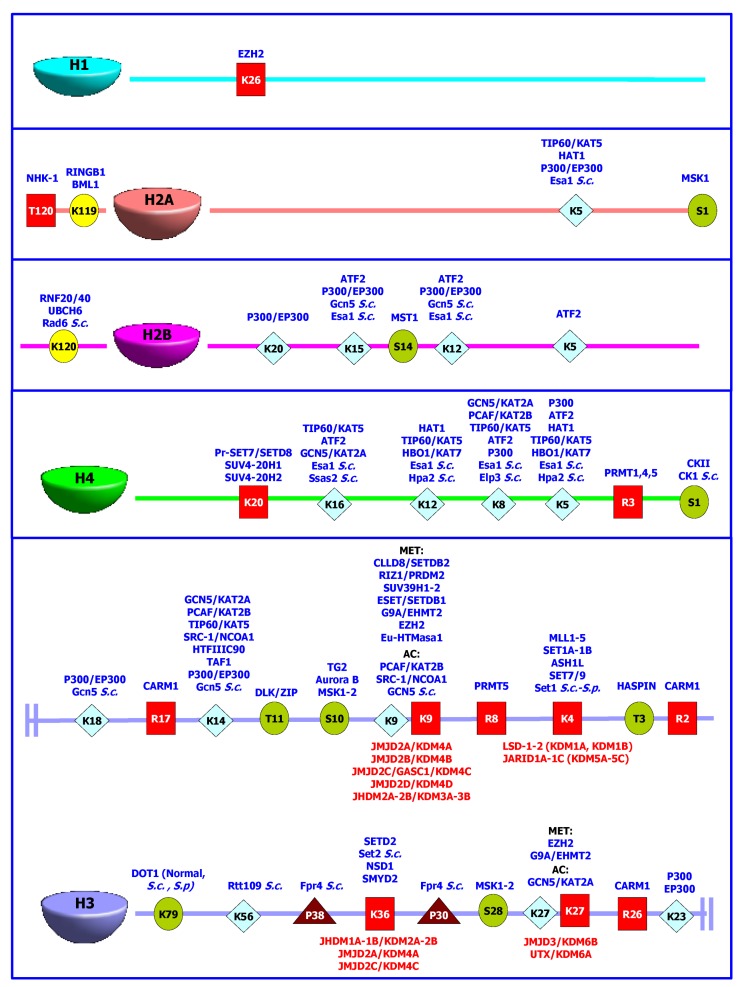
Post-translational modifications of histone proteins H1, H2A, H2B, H3 and H4 involved in epigenetic regulation. Target amino acids for isomerization (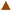), phosphorylation (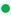), ubiquitination (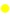), acetylation (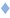) or methylation (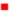) are shown as one-letter code, together with the different enzymes able to add (in blue) each functional group. The histone demethylases are shown in red, besides the lysine residues susceptible to their specific enzymatic activity.

At the functional level, genetic information resides inside the chromatin, which is a structure in the cell nucleus consisting of repetitive units of smaller structures called nucleosomes [2,3]. Nucleosomes consist of 146 base pairs (bp) of DNA wrapped in 1.75 turns around a histone octamer, all of which is organized as a central tetramer of H3/H4 histones surrounded by two histone H2A/H2B dimers [4,5]. The DNA between nucleosomes can be associated with a fifth histone, H1, to achieve the high-order compression of the chromatin [3,6]. At the structural level, two main chromatin conformations are distinguishable: euchromatin, with a low degree of compression associated with a “relaxed” conformation of the histone-DNA macro-complex, and heterochromatin regions, with a high degree of compression that can be accessory (susceptible to changes in the degree of compression) or constitutive (which is observed during the cell cycle) [3,6]. In epigenetics, variations in the structure or the degree of chromatin compression lead to the regulation of the genes associated with a specific genomic region because these variations facilitate or prevent access of the nuclear proteins required for processes such as gene expression [7]. In this manner, epigenetics regulates the reversible change from heterochromatin (compressed structure associated with the repression of gene expression) to euchromatin (structure accessible to the transcriptional machinery). This regulation occurs via mechanisms that involve DNA methylation and post-translational histone changes such as methylation, citrullination, and ADP-ribosylation of arginine (the latter modification also occurs at glutamic acid residues); methylation, acetylation, biotinylation, ubiquitination, and sumoylation of lysine; phosphorylation of serine and threonine; and the *cis*-*trans* isomerization of proline (Figure 1). Each of these modifications has a characteristic effect on the level of gene expression. For example, the addition of acetyl groups to the histones is frequently related to transcriptional activation, whereas deacetylation produces the opposite effect. Conversely, the methylation-demethylation of lysine can be associated with transcriptional activation or repression depending on the function of the residue and the degree of methylation [8,9].

In summary, these processes contribute to a finely regulated mechanism of control of genetic expression and are responsible for maintaining the cellular “equilibrium”. In fact, alterations in one or more components of such mechanisms can result in alterations in gene expression and/or cellular phenotype, which are the main causes of the emergence of several pathologies, including neurological diseases, diabetes, and diseases associated with endocrine dysfunction, as well as the different varieties of cancer that can affect human beings [10,11,12,13,14,15]. Consequently, major efforts have been directed toward the identification and characterization of different epigenetic regulators whose phenotypes have been altered in tumor cell lines with the goal of identifying potential therapeutic targets, including DNA-methyltransferases [16,17,18], histone acetyltransferases/deacetylases [17,19,20,21,22,23,24], and histone methyltransferases/demethylases [25,26,27,28,29], for the development of more efficient cancer treatment strategies.

The current work provides an updated description of a large family of histone demethylases that are responsible for maintaining the cellular phenotype by regulating histone methylation levels and serve as promising targets in the development of new treatments against a large variety of forms of cancer. New knowledge will be discussed in relation to the search for new anti-cancer targets, which could be targeted by a new generation of specific drugs directed against these altered histone modifiers.

## 2. Connection between Histone Methylation and Disease

**Figure 2:**
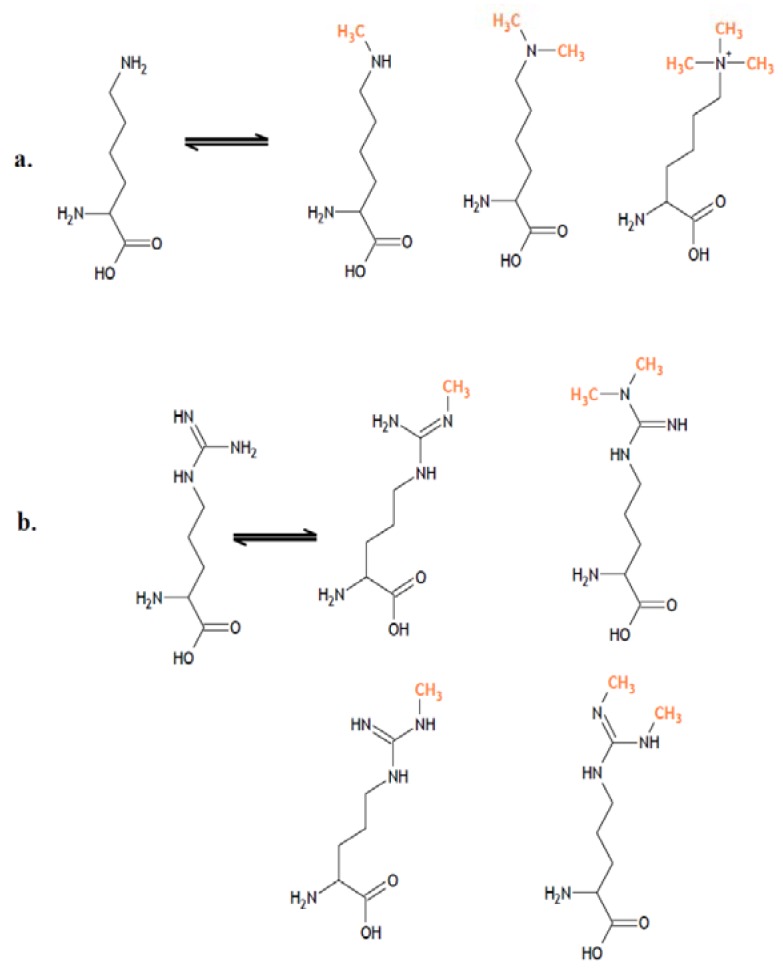
Methylation patterns of proteins at the lysine and arginine residues. In the histones, it is possible to find (**a**) mono-, di- and trimethylated forms of lysine, as well as (**b**) monomethylated and dimethylated forms for arginine.

In a large number of cellular processes, covalent modifications induced by methylation can affect different nitrogen-bearing amino acids, such as arginine, histidine, and lysine [30,31,32,33,34]. Histones can be mono-, di-, or tri-methylated at their lysine residues, and they can be mono- or di-methylated at arginine residues in a symmetric or asymmetric fashion (Figure 2).

Previously, histone methylation was considered to be a stable and irreversible mark of chromatin [35]. However, in 2004, Shi *et al*. described the mechanism of action of the first protein with specific demethylase activity against a lysine residue, LSD-1/KDM1A (lysine-specific demethylase-1), breaking the paradigm of irreversible methylation and opening a new research outlook on these proteins and their association with epigenetic regulatory mechanisms [36].

**Figure 3:**
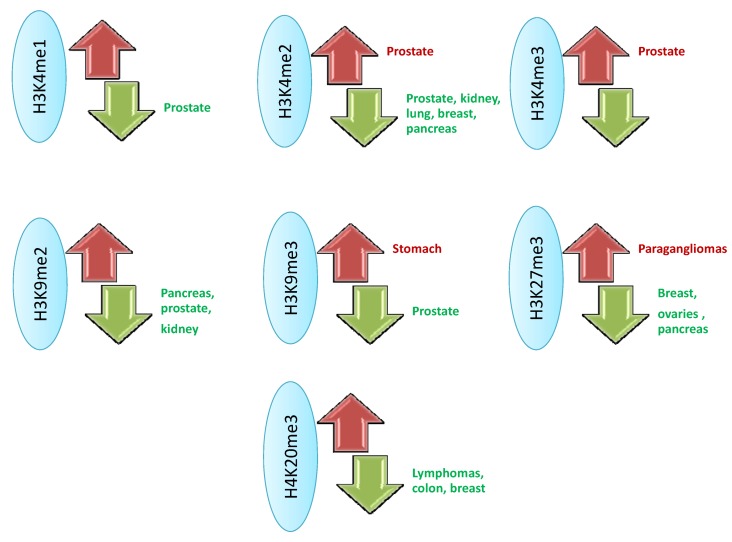
Pattern of methylation-demethylation associated with different types of neoplasia. The main alterations at the histone proteins H3 and H4 level are illustrated, highlighting the cancers associated with increase (red) or reduction (green) of methylation levels, facing normal cells [39].

The discovery of new sequencing and analysis technologies has allowed for the identification of the histone methylation pattern in the human genome [37], which, in turn, identifies associations between cellular phenotypes and methylation-demethylation as part of regular cell development and in processes related to the emergence of different pathologies. Inside the nucleosome, the H3 and H4 histones are the main targets of these modifications, which have been associated with the development of different pathologies, including cancer, that are associated with epigenetic defects (Figure 1 and Figure 3) [38,39]. Such observations have produced significant interest in the characterization of different proteins associated with the epigenetic regulation of histone methylation, among which the recently described histone demethylases are promising targets for future treatments against tumor cells.

## 3. Histone Demethylases and Cancer

**Figure 4:**
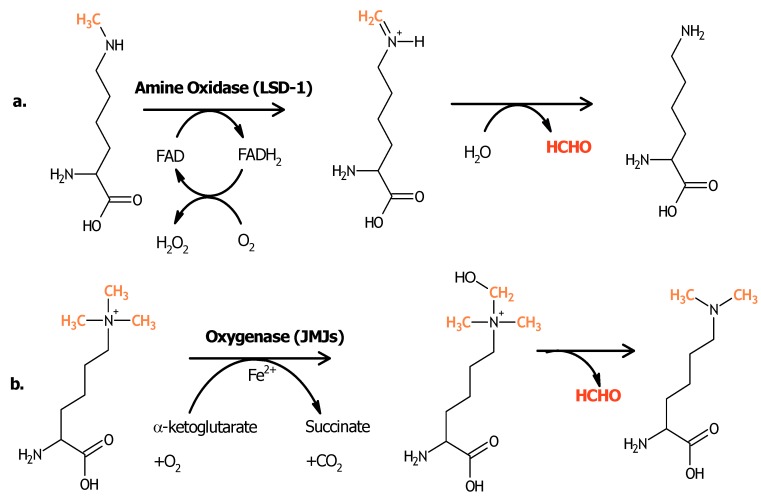
Mechanisms of histone demethylation [40]. (**a**) The proteins having an amine-oxidase FAD-dependent activity require at least one atom of hydrogen on the lysine’s amine group, implying these proteins’ activity on the mono- and dimethylated forms of this amino acid. On the other hand, those proteins with oxygenase activity (**b**) are able to add a hydroxyl group to the methyl group, in a Fe^2+^- and α-ketoglutarate dependent way, letting them act on the quaternary form of the methylated amine.

After the first characterization of a protein with demethylase activity, much effort has been made to identify and characterize other proteins with this activity in histone residues. The main proteins that have been discovered to date can be classified into two super-families: those belonging to the amine oxidase superfamily, which are dependent on FAD as a co-factor, and those corresponding to the oxygenase superfamily, in which the demethylase activity is dependent on Fe(II) and α-ketoglutarate. This latter superfamily is associated with the presence of a characteristic domain termed Jumonji (Figure 4). Among the most important histone demethylases associated with the development of cancer are LSD-1/KDM1A, and members of the Jumonji family JARID1A-1C/KDM5A-5C, JHDM1B/KDM2B, JMJD2C/KDM4C, JMJD2A/KDM4A, JMJD3/KDM6B, and UTX/KDM6A (Table 1), which will be analyzed in detail in the following paragraphs. 

**Table 1:** Histone demethylases and their association with cancer development.

Histone demethylase	Genebank Acc. Number	Alteration	Associated cancer	References
LSD-1/KDM1A	NM_015013	Over-expression	ER-negative breast, Prostate, Leukemia, Sarcomas, Lung, neuroblastoma	[31,41,42,43,44,45,46,47,48,49]
		Low expression	Hepatocellular, gastrointestinal, Hepatobiliary and metastatic breast carcinomas	[50,51]
JARID1A/KDM5A	NM_001042603	Over-expression	Gastric, Leukemia	[52,53]
JARID1B/KDM5B	NM_006618	Over-expression	Testicular, Ovaries, Breast, Prostate, leukemia	[54,55,56,57,58,59,60,61]
JARID1C/KDM5C	NM_004187.3 NM_001146702.1	Over-expression	Renal Carcinoma, Cervical	[62,63,64]
JHDM1B/FBXL10/KDM2B	NM_032590	Over-expression/Mutation	Lymphomas, Acute myeloid leukemia	[65,66,67]
		Low expression	Gliobastoma multiforme, brain	[68]
JMJD2C/KDM4C	NM_015061; NM_001146694; NM_001146695; NM_001146696	Over-expression	MALT and Hodgkin Lymphomas, myeloid leukemia, Breast, gliobastoma multiforme, prostate, desmoplasic meduloblastoma, sarcomatoid and esophageal carcinoma.	[69,70,71,72,73,74,75,76,77,78,79,80,81,82]
JMJD2A/KDM4A	NM_014663	Over-expression	Kaposi Sarcoma, Prostate, Breast, Colon, Bladder.	[18,47,83,84,85,86,87]
JMJD3/KDM6B	NM_001080424	Over-expression	Colon, Lymphomas	[88,89,90]
UTX/KDM6A	NM_021140	Mutation/Low expression	Advanced forms of cancer, Multiple mieloma, Leukemia, renal and bladder carcinomas.	[91,92,93,94,95,96]

### 3.1. LSD-1 and LSD-2

The LSD-1/KDM1A protein was the first demethylase protein to be identified [36]. In the mono- and bi-methylated forms, this amine oxidase presents specific activity on lysine 4 of histone H3 and forms part of an important macromolecular complex: CoREST [97,98,99]. LSD-1 is considered to be an important regulator of the formation and development of organs and tissues such as the heart, brain and skeletal muscle [100,101,102,103], together its essential role for cellular energy expenditure, inflammatory responses and hematopoiesis [104,105,106]. Interestingly, it has been reported that in the prostate, this enzyme can change its substrate to the mono-methylated form of lysine 9 as a result of its association with another important histone demethylase, JMJD2C. Together, both proteins have been associated with the development of prostate and bladder cancer through putative interactions with androgen receptors [41,43,47,48]. Additionally, the activity and overexpression of LSD-1 have been related to the emergence and development of different types of cancer, such as neuroblastoma [49], leukemia [31,42], sarcoma [44], lung [45], and ER-negative breast cancer [46]. In contrast, low levels of this protein have been observed in hepatocellular, gastrointestinal, hepatobiliary and metastatic breast carcinomas [50,51]. On the other hand, the understanding of molecular mechanisms ruling LSD-1 activity is in continuous evolution since its 3D structure determination [107], leading to the rational discovering of promising inhibitors with therapeutic potential [67,108,109,110,111]. Interactions based on these studies, LSD-1 is thought to be a potential marker for the early diagnosis and potential treatment of the malignant tumors mentioned above [112].

In addition, it has been recently described a new member of amine oxidase protein family: LSD-2/AOF1/KDM1B. Although this protein is homologous to LSD-1, it doesn’t participate in the chromatin-remodeling complexes as LSD-1 does [113,114]. This demethylase (showing specific activity for mono- and di-methylated Lys4 of histone H3) has been associated with the regulation of the inflammatory process mediated by NF-κB [115] and for establishing the maternal genomic imprints [116], as well as LSD-2 represents an important factor for induced pluripotent stem cell generation [117]. Although it has not been reported of any association for LSD-2 with human pathologies, the importance of its demethylase activity and LSD-1’s important as an anti-cancer candidate highlight the importance of continuing the functional and structural characterization of this protein [99,114,118].

### 3.2. JARID1 Family

Members of the JARID1 family of oxygenases recognize and act specifically on the bi- and tri-methylated lysine 4 of histone H3. One of the most representative members of this family is JARID1A/RBP2/KDM5A, which is an important regulator of the circadian rhythm [119,120]. This enzyme is also involved in cell cycle regulation [121] because it interacts with important proteins such as the retinoblastoma protein, and it is thought to affect pathways that are dependent on cyclins in some cancer types such as gastric cancer [52] and leukemia [53], as well as has been considered as a relevant marker to evaluate gliobastoma multiform survival [122]. Interestingly, it has been reported JARID1A is a critical factor for the development of transient drug tolerance in lung cancer cells [123], representing a key target to analyze how non-heritable aspects of cellular variability may significantly affect the evolutionary rate of cancer, in such way that initial drug treatments may be driving tumors to establish a genetically-based resistance by first selecting these non-heritable phenotypes generated by cellular or tissue variability [124]. On the other hand, the relevance of this protein has also been described in the emergence of other diseases including ankylosing spondylitis [125] and alopecia areata [126]. However, the current knowledge of the function of this protein at the cellular level is limited, although a role for this protein as a regulator of cellular growth via the Notch signaling pathway has been proposed [127], as well as it has been reported JARID1A and JARID1B are associated to the silencing of retinoblastoma target genes in senescent cells [128].

The second member of this family, JARID1B/PLU1/KDM5B has been recognized to be an important regulator of cell development and differentiation [129,130,131,132]. This enzyme has been strongly connected with the formation of different types of cancer because of the high expression levels found in leukemia, prostate, breast, testicular, and ovarian cancers, in which it can act as a repressor of tumor suppressor genes including BRCA1 or as a coactivator of growth and transcription factors such as TIEG1/KLF10 or the androgen receptor [54,55,56,57,58,59,60,61]. In summary, these results have fueled the search for inhibitors with anticarcinogenic potential directed against this protein [133].

Additionally, the third member of this protein family (JARID1C/KDM5C) is an important mediator during intellectual and cognitive development [134,135], and multiple defects in this protein have been associated with different pathologies including intellectual disability (mental retardation), short height, delay in speech abilities, and autism [135,136,137,138,139]. Similar to the other members of this family of proteins, JARID1C represents an interesting therapeutic target in the search for new drugs [140] because of its relevance and recent association with the development of renal carcinomas via the regulation of the von Hippel-Lindau tumor suppressor protein [62,64] and because of its implication in cervical cancer by serving as an oncogenic target of the human papillomavirus [63].

### 3.3. JHDM1B

JHDM1B/KDM2B, a member of the Jumonji protein family, exhibits oxygenase activity at the mono- and bi-methylated lysine 36 of histone H3. Although a role for this protein has not been defined at the cellular level, recent studies suggest a relationship between this methylase and important processes such as the regulation of cell proliferation and senescence [141,142] and the possible application of this protein as an enhancer in the reprogramming of stem cells [143]. Currently, this protein is considered to be a candidate tumor suppressor gene because its expression levels are strongly reduced in different forms of cancer, including acute myeloid leukemia, lymphoma, and glioblastoma multiforme, the most aggressive form of brain cancer [65,66,67,68,144,145].

### 3.4. JMJD2C

JMJD2C/KDM4C is a histone demethylase that exhibits oxygenase activity at the bi- and tri-methylated lysine 9 in histone H3 [69], and this protein is involved in the development and self-renewal of undifferentiated and embryonic stem cells, the regulation of adipogenesis, and hypoxia responses [146,147,148,149]. In addition to these functions, JMJD2C has been associated with the emergence of different pathologies such as alopecia areata and autistic spectrum disorders [126,150]. It is also considered to be an important amplified oncogene in different types of cancer such as sarcomatoid and esophageal carcinomas [70,82], myeloid leukemia [71,72], lymphoma [73,74,80], breast carcinoma [75,76], desmoplastic medulloblastoma [81], and glioblastoma multiforme [77,78]. Although the mechanisms by which this protein is involved in the development of these types of cancer have not been fully understood, the characteristics of this protein have suggested JMJD2C as a potential candidate for the development of specific treatments against these forms of cancer [25,110,151].

### 3.5. JMJD2A

JMJD2A/KDM4A, another member of the Jumonji protein family, is associated with the transcription of cell proliferation genes due to its capacity to bind to chromatin-modifying and cell cycle regulator proteins [152]. After the establishment of its methylase activity on bi- and tri-methylated lysines 9 and 36 of histone H3 [153], JMJD2A has been shown to play important roles in skeletal muscle cell and neural crest differentiation, the maintenance of maternal stem cells, and DNA repair [148,154,155]. This protein is related to the development of diseases such as cardiac hypertrophy [156], alopecia areata [126], and different forms of cancer including Kaposi’s sarcoma associated with herpesvirus [85], prostate cancer (via regulation of the activity of the androgen receptor) [83,84], breast cancer [86], colon cancer [87], and bladder cancer [47]. Such results highlight the relevance of this protein at the therapeutic level, and have arised the interest to determine JMJD2A’s mechanism of action and specificity based on its three-dimensional structure [157,158,159,160,161]. This strategy may lead to the identification and characterization of specific inhibitors with therapeutic potentials [25,151,162,163]. However, knowledge of the function of JMJD2A in other carcinogenic lines and the determination of its role as a therapeutic anti-cancer target still remain to be established.

### 3.6. JMJD3

JMJD3/KDM6B, another member of the Jumonji family, exhibits high specificity for the bi- and tri-methylated lysine 27 residue of histone H3 [164], and it serves as a part of important multiprotein complexes known as MLL3 and MLL4, which are associated with the regulation of the activity of Polycomb family proteins [165,166,167,168,169]. Such characteristics have promoted the investigation of the relevance of this protein at the cellular level, allowing for its identification as a regulator in endothelial, endodermal, osteoclast, macrophage, and neural lineage differentiation [170,171,172,173,174]. Additionally, the influence of this enzyme on the occurrence of a large number of diseases has been described, with implications in systemic lupus erythematosus [175], vasculitis associated with antineutrophil cytoplasmic antibodies (ANCA) [176], colon cancer [90], and lymphoma [88,89]. Although the role of this protein in these pathologies has not been fully described, JMJD3 is associated with the activity of different oncogenes such as NPM-ALK and BCL2, or tumor-suppressing genes such as BTG3 [89,177,178]; justifying the inclusion of this protein in the search for new anti-cancer epidrugs [179].

### 3.7. UTX

Similarly to JMJD3, UTX/KDM6A acts on the mono- and di-methylated forms of lysine 27 in histone H3 [180] and is also a part of the MLL3/MLL4 complexes [166,167]. UTX was the first histone demethylase known to be associated with cancer as a result of specific mutations [96]. In combination with the relevance of UTX in cellular differentiation at the cardiac, hematopoietic, sperm, brain, and muscular levels [135,181,182,183,184], this enzyme is also a tumor suppressor gene candidate (like JHDM1B). This designation was the result of multiple investigations that identified mutations or inactivating events associated with the differentiation of tumor cells in multiple myeloma [94,96], acute lymphoblastic and chronic myelomonocytic leukemias [93,95], bladder carcinoma [92], and renal cell carcinoma [91]. Similarly to JARID1A, UTX has been recently linked to regulation processes that are dependent on the retinoblastoma protein, as it is primarily associated with control of the expression of different factors that are part of the pathways that are dependent on this important enzyme [185,186]; this activity is related to the potential role of this protein as a tumor suppressor [187].

## 4. Therapeutic Approaches

**Figure 5:**
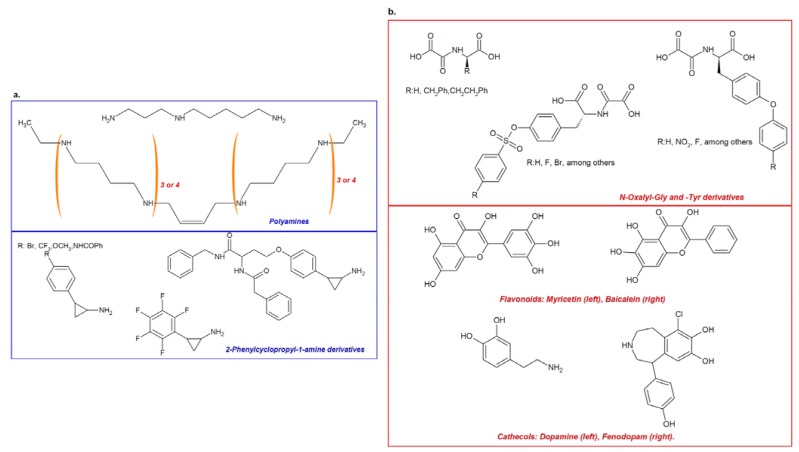
Inhibitors of histone demethylation. Specific inhibitors for LSD1 can be designed according to the similarity of this demethylase’s substrates with the FAD-dependent polyamine oxidases targets (**a**, **top frame**), as can be observed for spermidin-like inhibitors, octamines (n = 3) or decamines (n = 4). Another pathway for designing amine oxidases’ inhibitors involves their mechanism of activity, targeting FAD by radical oxidation reactions with “suicide” inhibitors containing a phenylcyclopropylamine core (**a**, **bottom frame)**. Histone demethylases with oxygenase activity can be inhibited by using α-ketoglutarate analogues, as these could be able to bind to Fe^2+^. Some of the most promising epidrugs includes some derivatives of *N*-oxalyl glucose and *N*-oxalyl tyrosine, (**b**, **top frame**), in conjuction with the recent description of flavonoids and chatecols showing competitive and non-competitive inhibition, possibly associated with these natural product’s ability to bind iron (**b**, **bottom frame**).

Recently, an indirect or direct association has been established between histone demethylases and the emergence and progression of cancer. Among the potential strategy for targeting KDMs, the identification of molecules with the capacity to specifically inhibit the enzymatic activity of these proteins represents a promising path for treatment [10,188]. As the first member of this important family of proteins, LSD-1 represents an important target for the so-called “epi-drugs”. Among the molecules with pharmaceutical potential are oligoamine analogs, originally designed based on the significant similarity between LSD-1 and other FAD-dependent polyamine oxidases (Figure 5a, top panel) [109,189], and specific inhibitors possessing a common 2-phenyl cyclopropyl-1-amine nucleus [108,118,190,191]. These specific inhibitors for LSD-1 were designed based on the mechanism of action of the enzyme (which is similar to that of monoamine oxidases), and are able to reach inhibition constants in the micromolar range (Figure 5a, lower panel) [108,118,190,191].

These findings have promoted the search for new inhibitors and have resulted in the recent description of namoline, a gamma-pyrone capable of inhibiting LSD-1 and altering the growth of prostate cancer tumor cells [192]. The first generation of LSD-1 inhibitors, which are of the tranylcypromine type, exhibited low potency and selectivity. After the search was shifted to focus on chemical structures that target the active site of the protein, selectivity was increased [193]. *In vitro* and *in vivo* studies using nanomolar concentrations of phenocopied, a tranylcypromine analog and inhibitor of LSD-1, demonstrated a pro-apoptotic effect in primary acute myeloid leukemia (AML) cells without affecting the repopulation potential of hematopoietic stem cells and progenitor cells [194]. These data suggest that LSD-1 is a key protein for the development of selective therapeutic targets for leukemia.

In the same manner, the inhibition of LSD-1 can increase the sensitivity of promyelocytic leukemia (APL) to the treatment with all-trans-retinoic acid (ATRA). Usually ATRA loses its activity in APL, and the cause of this alteration appears to be a reduction in the methylation of lysine 4 of histone H3. Under such conditions, the inhibition of LSD-1 can facilitate the activity of ATRA in APL cells, as it has been validated using the LSD-1 inhibitor trans-2-phenylcyclopropylamine [195].

In addition, iron- and α-ketoglutarate-dependent histone demethylases are also targets of important studies, allowing for the identification of inhibitors designed on the basis of the mechanism of the unique activity of these enzymes. Among the most important inhibitors, several molecules that are analogous to α-ketoglutarate have been described. These inhibitors range from molecules designed using *N*-oxalylglycine or *N*-oxalyltyrosine as a base molecule [25,162,196] (Figure 5b, upper panel) to natural products such as flavonoids and catechols that were identified as a result of screening libraries of pharmacologically active compounds [197] (Figure 5b, lower panel). Demethylase inhibitors for Jumonji-containing domain proteins usually compete with the 2-oxoglutarate factor and bind to the catalytic region containing iron. Compounds that possess high polarity, such as 2,4-pyridine-decarboxylases, inhibit the activity of Jumonji by interfering with its enzymatic activity, in the same way other 2-oxoglutarate-like molecules inhibit the HIF prolyl hydroxylase-1 oxygenases HPH1/EGLN2 and HPH2/EGLN1.Interestingly, two new series of jumonji demethylase inhibitors, 8-hydroxyquinolines (e.g., SID 85736331) and 2,2-bipyridines, are potent inhibitors with higher selectivity and better pharmacological properties than those exhibited by other inhibitors. The structures of these two new types of compounds make them promising therapeutic alternatives that could be pharmacologically enhanced for better penetration into the cell interior [198,199].

These results clearly highlight the potential of the histone demethylase superfamily members as therapeutic targets against the diverse neoplastic diseases that affect human beings. There is support for structural analyses to increase the understanding of the spatial enzyme-inhibitor interaction [99,200,201] and, hence, promote the specific molecular design of a new generation of epi-drugs targeting members of the amine oxidase superfamily [108,202,203] or those belonging to the oxygenase family [25,159,162].

## 5. Conclusions

Regulation of gene expression encompasses a complex network of molecular interactions that define the cellular phenotype of tissues and organisms. Alteration on the epigenetic balance can result in the emergence of terminal diseases, among which are the several forms of human cancer. Although, much work remains to be taken in the search for new strategies against the different types of cancer, in recent years, significant advances have been made in the identification and characterization of new potential targets for the development of specific therapies to treat multiple forms of cancer. Together with the continuous discovering of new macromolecules associated with human neoplasm, the recent discovery of histone demethylases has provided a new perspective in the search for anti-cancer therapeutic targets due to their unique enzymatic activity and their association with the cellular phenotype of a large number of human tumor lines.

Either as early markers or as anti-tumor candidates, histone demethylases currently represent a promising group of macromolecules that increase our understanding of malignity and the mysteries of their formation, development and evolutionary progress. LSD1 was the first demethylases cloned and perhaps the most promises protein as therapeutic target. Recently evidences showed that LSD1 played an important role in a broad spectrum of biological processes, including cell proliferation, adipogenesis, spermatogenesis, chromosome segregation and embryonic development. Furthermore, LSD1 also could promote progress of tumor by inhibiting the tumor suppressor activity of p53. To date, as a potential drug for discovering anti-tumor drugs, the medical significance of LSD1 inhibitors have been greatly appreciated. However, it is necessary to be attentive when analyzing and applying this new knowledge, as relevant targets as LSD-1 could be observed as an oncogene [31,41,42,43,44,45,46,47,48,49] or a tumor-suppressor [50,51], in an apparent situation-dependent biological role for this protein. These observations, enhanced the importance of histone demethylases for cellular and tissue phenotype maintenance as part of macro-complexes [51,114,127,152,169], must be taken into account to overcome any side effects facing their use as therapeutic targets [106]; thus highlighting the importance of improving our comprehension about biological and molecular role of histone demethylases, to be established as potential targets for a new generation of epigenetic drugs directed against their enzymatic activity. Various demethylases from Jumonji family are in preclinical studies for cancer including prostate cancer, glioblastoma, breast cancer, and diverse kind of leukemia.

In summary, this report is part of the future on the continuous fight against cancer; searching for new specific therapeutic strategies based on the understanding of the epigenetic regulators discussed herein and those different molecules rationally designed against these specific targets; the epi-drugs.
